# Evaluating adherence to patient registration paperwork guidelines: a mystery shopper study in English primary care

**DOI:** 10.1136/bmjopen-2025-100719

**Published:** 2025-11-11

**Authors:** Nathan Hodson, Onyedikachi Oluferanmi Onyeaso, Stuart Mills, Cass R Sunstein, Wändi Bruine de Bruin

**Affiliations:** 1 Warwick Medical School, Coventry, UK; 2 USC Sol Price School of Public Policy, Los Angeles, California, USA; 3 Coventry and Warwickshire Partnership NHS Trust, Coventry, England, UK; 4 Leeds University Business School, Leeds, England, UK; 5 Harvard Law School, Cambridge, Massachusetts, USA; 6 Schaeffer Institute of Public Policy and Government Service, USC Sol Price School of Public Policy, Los Angeles, California, USA

**Keywords:** Health policy, Primary Care, Information technology, Health Services Accessibility

## Abstract

**Abstract:**

**Objective:**

To evaluate adherence to National Health Service (NHS) patient registration ID guidelines among General Practitioners’ (GP) practices.

**Design:**

A mystery shopper study, including website reviews and phone calls.

**Setting:**

Rural and urban parts of the United Kingdom’s West Midlands.

**Participants:**

85 randomly selected GP practices.

**Primary and secondary outcome measures:**

In January–April 2024, GP’s websites were reviewed before phone calls in which our ‘mystery shopper’ was asked to register without photo ID and proof of address.

**Results:**

Of 85 GP practices, 60 (71%) breached NHS guidance either online or over the phone, with only 25 (29%) consistently following NHS guidance. Phone calls to rural (vs urban) GP practices were more likely to yield refusal of registration without photo ID and proof of address, despite rural (vs urban) GP practices making similar statements online. During some phone calls, practices sought to negotiate a compromise by requesting less robust ‘documentation’, such as an addressed parcel.

**Conclusions:**

GP practices commonly refuse registration to people without photo ID or proof of address, thus creating ‘sludge’ and undermining access to healthcare especially for poor, vulnerable patients, including immigrants. Changing GP practices’ websites would not address this problem if erroneous information is still provided over the phone. GPs and practice managers should ensure that all staff follow NHS guidance to allow registration without these documents.

**STRENGTHS AND LIMITATIONS OF THIS STUDY**

- This mystery shopper study combines two modes of evaluating General Practitioners’ practice registration: website reviews and phone calls.
- The experience of somebody trying to register with a GP in the UK without a photo ID or proof of address was measured directly.
- Several practices were unreachable by phone.
- GP practices were not visited in person.

## Background

 Universal access to healthcare is a founding principle of the National Health Service (NHS).^1^ Requiring photo ID or proof of address can exclude people from care, especially low socioeconomic status people as well as immigrants.^2^,^5^ General Practice (GP) is the main point of entry to NHS care, and so the NHS Policy and Guidance Manual states that inability to provide photo ID or proof of address requirements should not prevent people from registration with a GP surgery. The NHS Policy and Guidance Manual states, ‘If a patient cannot produce any supportive documentation but states that they reside within the practice boundary, then practices should accept the registration’ because many people are ‘legitimately unable to produce any of the listed documentation’.^2^

‘Sludge’ describes barriers to accessing public services, including ID requirements and paperwork burdens.^6^ These barriers impose a ‘time tax’ and may even make it impossible for some to obtain access to such services. When GP practices unnecessarily require photo ID or proof of address for patient registration, they are creating sludge in contradiction to NHS guidance.^7^

There are 280 000 families in the UK who live in temporary housing and potentially lack current proof of address.^8^ About 22% of white British people and 45% of black British people over 17 years old have no driving licence.^9^ Moreover, 8 million people in the UK (13.5%) have no passport from any country.^10^ In 2023 alone, 1.2 million people immigrated to the UK and may therefore be uncertain about their ability to register with a GP.^11^

Healthwatch groups have raised concerns that GP practice policies violate NHS guidance about patient registration.^12 13^ When the charity Doctors of the World UK tried to help immigrants and other vulnerable people to register with a GP practice, they found that requests for photo ID and proof of address were widespread.^14 15^ In most cases, however, Doctors of the World UK was able to successfully intervene and negotiate patient registration.^5 15^ Evidence is needed about how many people get refused when they try to register without photo ID and proof of address on their own, without the intervention of Doctors of the World UK. It is also important to evaluate the information that GP practices provide about paperwork requirements in different modalities, such as online and over the phone. While Doctors of the World UK was able to negotiate registration over the phone,^14 15^ there is a concern that GP practice staff may create paperwork requirements and registration hurdles to prevent people without documentation from accessing finite NHS resources.^16^

## Aims

Following concerns that people without photo ID or proof of address may experience barriers to patient registration,^12 13 15 16^ we aimed to evaluate adherence to NHS patient registration paperwork guidelines among General Practitioners’ (GP) practices. GP practice websites were reviewed before phone calls in which the first two authors posed as ‘mystery shoppers’ asking to register without photo ID and proof of address. Such a ‘mystery shopper’ approach is designed to examine how people are treated^17^,^21^ and may identify registration barriers or ‘sludge’ faced by people without paperwork.^7 22 23^ Specifically, we examined: (1) What percent of GP practices refused registration without photo ID and proof of address, online and over the phone, in violation of NHS guidance?; (2) What percent of GP practices refused registration without photo ID and proof of address when phoned, in violation of NHS guidance?; (3) Was information given on websites consistent with information given over the phone? and (4) What information were callers given when they were refused registration?

## Methods

### Sample

We randomly sampled 100 GP practice websites from the West Midlands, including 50 from urban multicultural Birmingham and Solihull and 50 from Worcestershire and Herefordshire.^5^ Of 100 GP practices sampled, four had no website and three had no information for new patients on their websites. When the remaining 93 practices were contacted over the phone, eight did not answer the phone after four attempts, as detailed below. Therefore, 85 practices were included in the final sample: 39 (46%) from Birmingham and Solihull, and 46 (54%) from Herefordshire and Worcestershire.

### Website review

Each GP practice website was evaluated by one researcher in January 2024. GP websites were coded as breaching NHS guidance if they stated that photo ID or proof of address was required. They were coded as not breaching NHS guidance if they explicitly stated that photo ID or proof of address was not required, or if they did not make any statement about paperwork requirements. To test and develop our coding scheme (online supplemental appendix 1), the first two authors independently evaluated the websites of 10 GP practices not included in the present study. They reached 100% agreement.

### Phone script

The first two authors conducted phone calls in January-April 2024, following the ‘mystery shopper’ approach that is designed to examine how people are treated.^17^,^1924 25^ One had a local English accent from the West Midlands and the other had a Nigerian accent, which did not affect whether photo ID or proof of address were requested (*χ^2^*(1)=0.01, p = 0.91). Like the GP websites, phone calls were coded to reflect whether GP practices permitted registration without photo ID or proof of address, consistent with NHS guidance (online supplemental appendix 2). We also recorded explanations and the advice GP practices gave when declining registration.

To develop a phone script, the first two authors made pilot phone calls and listened to each other’s conversations (online supplemental appendix 2). The phone script stated that the ‘mystery shopper’ had recently moved to the country from Africa. They stated their desire to register, but that they had no photo ID because it was with the Home Office and had no proof of address because they had recently moved in with their girlfriend. Indeed, these are common reasons for not having photo ID or proof of address for patient registration.^15^ Mystery shoppers did not refer back to the website or the NHS guidance during the conversation.

Phone calls were conducted in the afternoon when most GP practices are less busy. Each practice was contacted on three different days until they answered. On each day, mystery shoppers waited on hold for a maximum of 5 min. If the phone remained unanswered for 5 min on three instances, they phoned for one more instance, waiting up to 20 mins. After each hour of phone calls, the mystery shoppers conferred and checked whether they agreed on coding.

### Analysis

To answer our first and second research questions, we reported on the percent of GP practices that required photo ID or proof of address online or over the phone, and conducted a χ^2^ test to examine whether the percent differed by urban or rural location. To answer our third research question, we reported a χ^2^ test to examine whether NHS guidance compliance was consistent between websites and phone calls. Subgroup analyses were conducted but should be interpreted with caution due to low numbers. Stata-16 was used to conduct analyses for the first two research questions. To answer our fourth research question, we reported information provided to mystery shoppers over the phone.

### Ethics

Ethical approval was provided by the Institutional Review Board of the University of Southern California. The deception inherent to this mystery shopper study was ethically defensible because (1) non-deceptive research methods, such as interviews or surveys with practice staff may have under-estimated violation of NHS guidance due to social desirability bias, (2) access to healthcare is critical, especially among vulnerable populations without photo ID or proof of address, (3) we maintained the confidentiality of GP staff and (4) the time burden on GP staff was kept to a minimum.^20^

### Funding

The study was conducted without funding.

### Patient and public involvement

There was no direct patient and public involvement in the design or interpretation of this study.

### Data sharing plan

Data are available on reasonable request.

## Results

### What percent of GP practice websites refused registration without photo ID and proof of address, in violation of NHS guidance?

**Figure 1:**
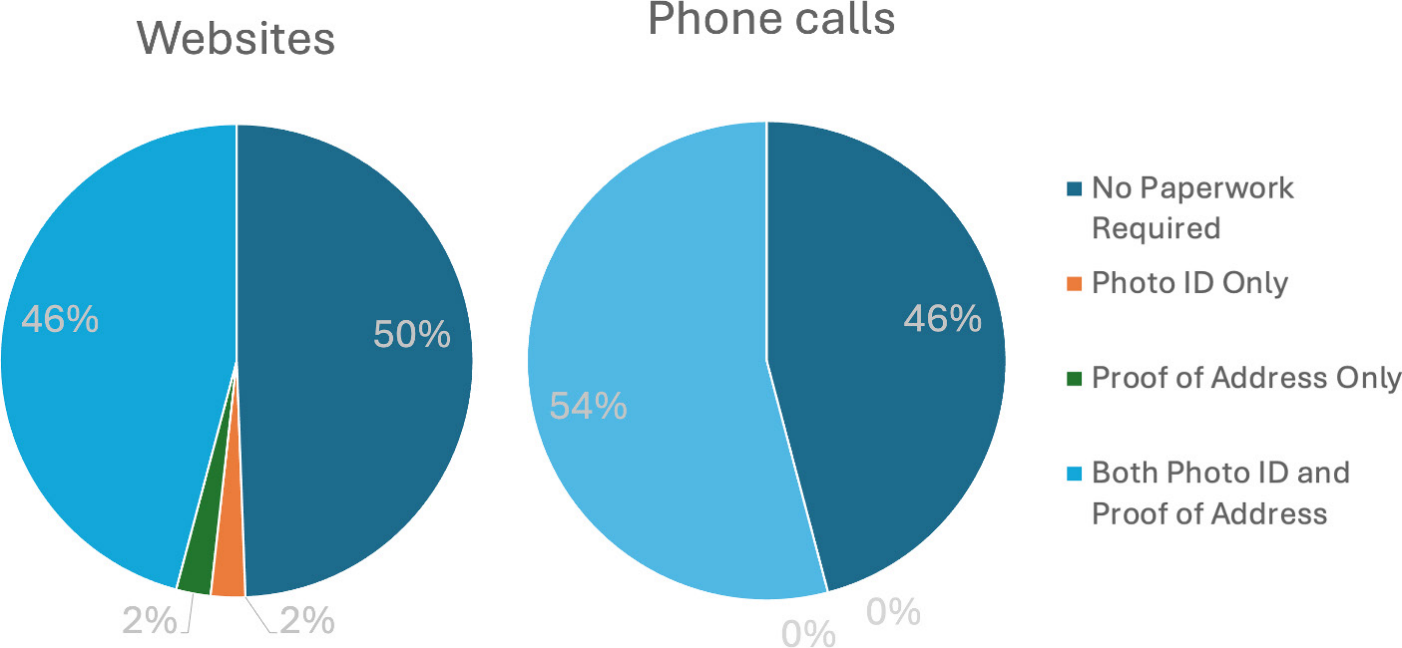
Percent of clinics imposing patient registration requirements on their websites and over the phone

Of 85 GP websites, only 42 (49%) followed the NHS guidance and stated that they required no photo ID and proof of address. Additionally, 39 (46%) websites required both photo ID and proof of address, 2 (2%) required only photo ID and 2 (2%) required only proof of address (figure 1). Overall, 28 (61%) GP websites of rural Herefordshire and Worcestershire practices and 15 (38%) GP websites of urban Birmingham and Solihull practices required at least one form of documentation (χ^2^(1) = 4.24, p = 0.03).

### What percent of GP practices refused registration without photo ID and proof of address over the phone, in violation of NHS guidance?

At 46 (54%) of 85 practices, the mystery shopper was refused registration due to lack of photo ID or proof of address, contradicting NHS guidelines and imposing sludge. At only 39 (46%) of 85 practices, the mystery shopper was advised by phone that he could register without photo ID or proof of address, following NHS guidelines. Advice given by phone did not differ between the rural Herefordshire and Worcestershire regions and the urban Birmingham and Solihull regions (χ^2^(1) = 0.70, p = 0.15).

### Was information given on websites consistent with information given over the phone?

**Figure 2:**
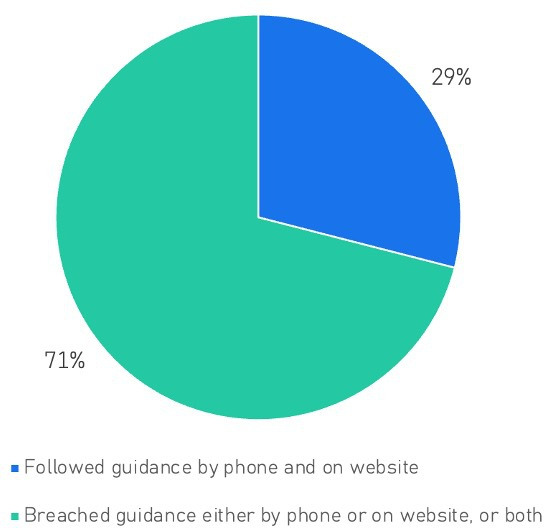
Percent of clinics following NHS patient registration guidance both online and over the phone (vs. Not)

Of 85 GP practices, 60 (71%) breached NHS guidance either online or over the phone, with only 25 (29%) consistently following NHS guidance (figure 2). GP practices whose websites required photo ID or proof of address were also more likely to require proof of address and photo ID over the phone (*χ^2^*(1) = 6.22, p=0.01), but inconsistency was still common (table 1). At 17 (20%) GP practices, information provided by phone contradicted websites that did not require photo ID or proof of address. At 14 (16%) GP practices, information provided by phone contradicted websites that did require photo ID or proof of address. Rates of consistency between website and telephone advice did not differ between the Birmingham and Solihull region and the Herefordshire and Worcestershire region (χ^2^(1) = 1.57, p=0.21).

**Table 1:** Number (%) of clinics following NHS patient registration guidance on their websites vs. over the phone

Phone call breached NHS guidance	Phone call followed NHS guidance
Website breached NHS Guidance	29 (34%)	14 (16%)
Website followed NHS Guidance	17 (20%)	25 (29%)

Number (percent of total) is shown in each cell.

NHS, National Health Service.

### What information were callers given when they were refused registration?

The phone calls revealed a high degree of uncertainty and ambiguity around policies. At three practices, callers were put on hold while the receptionist clarified the policy with a colleague.

Several GP practices helped callers to identify valid documentation, and sometimes internal policies were relaxed or bent. Typically, practices which allowed registration without photo ID or proof of address initially asked for proof of address or photo ID, but then relented, acknowledging that they were not able to refuse registration on that basis. Since the mystery shopper stated that the caller had recently moved in with his girlfriend, three practices asked for the girlfriend to vouch for the caller’s address either in person or in writing and two suggested bringing in proof of address with the girlfriend’s name instead. At three other practices, callers were asked to bring any parcel with their name and address. One said they would accept a letter showing an address in another country. At four practices, the caller was advised that they could submit a registration form without photo ID and proof of address and the administrative staff would make a final decision about whether to process the registration. At 13 practices, the callers were simply advised to wait until some form of photo ID or proof of address had arrived.

At four GP practices, the caller was advised to contact other local practices that did not require photo ID or proof of address. At one other GP practice, the caller was advised to phone 111 and at yet another advised to go to urgent care.

Comments were also made about the caller being from another country. At one GP practice, the proof-of-address policy was justified by noting ‘the system can be abused’. Elsewhere, a Biometric Residence Permit was demanded. At another, the caller was asked, ‘How did you get into the country then?’ when he shared, according to the script (online supplemental appendix 2), that his passport was currently with the Home Office. Although there was no statistical difference in requests for photo ID or proof of address between the two mystery shoppers, these comments were made only to the caller with a Nigerian accent, and not to the one with an English accent. At some other practices, the caller was warmly welcomed to the UK.

## Discussion

NHS guidance states that patients should not be refused registration at a GP practice on the basis of their inability to provide proof of address or photo ID. We selected 100 GP practices in the West Midlands, reviewed 93 of their websites and reached 85 by phone. 71% violated NHS guidance by introducing sludge either via their website or when phoned. 36% of practices provided information by phone which differed from information on the practice website.

### Strengths and limitations

This study brings together two modes of evaluating sludge in GP practice registration: website review and mystery shopper phone calls. Practices were exclusively from the West Midlands. Results are similar for practices in London.^5^ Several practices were unreachable by phone, which is a well-known problem, leaving a gap in our knowledge that could be addressed by an in-person mystery shopper study.^21^ Such a study could also validate whether information provided via websites and phone calls is followed in person.

### Comparison with the literature

This is the first study to reveal the inconsistent information prospective patients face when trying to register with a GP practice while lacking photo ID or proof of address. Previous research had raised concerns that people without photo ID or proof of address need the intervention of Doctors of the World UK to get registered,^14 15^ and that some GP staff deliberately create barriers to registration as a means of rationing care.^5 16^ This study shows how commonly people without photo ID or proof of registration get refused when they try to register on their own, and how inconsistent the information is that they receive.

Indeed, we found that people without ID or proof of address may receive inconsistent information across websites and phone calls. Even when GP websites state that registration is allowed without photo ID or proof of address, registration may be refused over the phone. Thus, practices are publicly stating a sludge-free policy online while implementing an exclusionary policy over the phone, perhaps unbeknownst to GPs and practice managers. This sludge is opaque and difficult for activists and scholars to challenge, given the most common way of evaluating policies has been through website reviews.^5 12 13^

Sludge of this kind is problematic because it keeps vulnerable populations from accessing healthcare. Doctors of the World UK notes that immigrants are one vulnerable group being turned away due to lacking paperwork, in addition to people experiencing homelessness.^15^ While our findings do not reveal any intent on the part of GP practice staff to single people out by race, the widespread requirement for photo ID and proof of address may exclude many more Black British people than White British people.^9^ People with epilepsy may also lack photo ID due to having had their drivers’ licences taken away, yet they need registration in order to be able to access management of their epilepsy.^26^

Although the NHS Policy and Guidance Manual states that GP practices may ‘apply a consistent but non-discriminatory policy to ask for patient ID’ during the registration process (Part B 4.9.4), it is followed up with the statement that ‘If a patient cannot produce any supportive documentation but states that they reside within the practice boundary then practices should accept the registration’ because many people are ‘legitimately unable to produce any of the listed documentation’.^2^ What this study measured was whether registration was possible without photo ID or proof of address, not whether photo ID or proof of address was requested. Indeed, in cases where photo ID or proof of address was requested but not required and registration was possible without it, the practice was recorded as in keeping with NHS guidance.

To be sure, the NHS, especially primary care, is under extraordinary pressure. But in this context, refusing registration to people without documentation is not an acceptable way to ration care, as NHS England itself has said.^27^ Not only does that practice exclude vulnerable populations, but the additional administrative work involved means that even among people who have all required paperwork, there is a risk that healthcare will not be accessed or delayed.^28^ Alternatively, the patient may take the advice given at several practices and use 111 or urgent care instead, meaning their care costs the NHS more and lacks the continuity primary care provides. Interventions to tackle this sludge could realise efficiencies in primary care provision while expanding primary care coverage.

### Implications for research and for practice

This study’s findings suggest GP partners and practice managers should keep in mind that, whatever information is written on the website, an exclusionary policy may be implemented by GP staff when prospective patients contact the practice. Even when the practice website states that people can register without photo ID and proof of address, prospective patients can still be turned away for lacking photo ID or proof of address. It is possible that in many domains, involving healthcare and beyond, people face more sludge, and hence more barriers, than relevant guidance authorises, and also more sludge, and hence more barriers, than relevant websites state is required. ‘Street-level’ sludge, not authorised by law or suggested by relevant guidance, and not identified publicly, may be pervasive, and may affect vulnerable populations in particular.^29^

It is therefore important that practice leaders clearly communicate expectations that all staff must follow the NHS Policy and Guidance Manual.^2^ Practice leaders may be the only people able to change front-line policy of so-called ‘street-level bureaucrats’, and we call on them to take action.^29^

While this study identifies the sludge problems that prospective patients face when registering with a GP practice, future scholarship should explore effective means of ‘desludging’ this process.^27^ Our results demonstrate that the NHS Policy and Guidance Manual alone is insufficient to change practice.^2^ Further action is required to implement it; the ‘de-implementation’ literature from implementation science provides one potential avenue for structuring such attempts at policy change.^30^ Mystery shopper studies may also drive policy changes themselves.^31^ Removing registration sludge is the first step to ensuring that everybody in the UK has equal access to the personalised and efficient care provided in primary care.

## Supplementary material

10.1136/bmjopen-2025-100719 online supplemental appendix 1

## Data Availability

Data are available upon reasonable request.
