# Supplementary material for: Evaluating adherence to patient registration paperwork guidelines: a mystery shopper study in English primary care
Source: BMJ Open. 2025 Nov 11;15(11):e100719. doi: 10.1136/bmjopen-2025-100719 (PMC13059835; doi:10.1136/bmjopen-2025-100719)
Supplement: online supplemental appendix 1 [file bmjopen-15-11-s001.docx]

**Appendix 1**

| New patient page on website? |  |
| --- | --- |
| Suggests the NHS website |  |
| Multiple different forms must be completed (1/0) |  |
| "How many forms? (number)" |  |
| Refugees only |  |
| New Patient Form other than GMST1 |  |
| Can fill form in the practice |  |
| Cannot fill form in practice |  |
| ""NHS card" or “medical card” |  |
| Total ID required (excluding abroad) |  |
| No examples |  |
| Passport |  |
| Drivers licence |  |
| Provisional DL |  |
| Other? |  |
| Must be valid |  |
| ""ID is not mandatory""" |  |
| quote for ID not mandatory" |  |
| Total Proof Address" |  |
| Must be recent (1/0) |  |
| Bank statement |  |
| Utility Bill |  |
| Council Tax |  |
| Tenancy agreement |  |

**Appendix 2**

I would like to register and I want to find out more about more about the process

(Expected response - look on website)

I looked on the website but I had some questions

(ok tell me the questions)

I'm worried I don't have the right documents - is it ok for me to register without bringing documents?

*if they say no documents needed then thank them and hang up*

*if they say you do need documents*

I have moved in with my girlfriend this week so I dont have any bills at this address yet, and I won't get any until the end of the month. My bank account will arrive in one month. Can you register me today anyway?

Thanks or... OK so I will have to sort that out

I don't have any photo ID, because my passport has expired and I haven't done my driving test yet or got a licence. Can you register me today anyway?

Thanks or... OK so I will have to sort that out

I'm from abroad and my passport and paperwork is with the home office at the moment

Thanks or... OK I will have to sort that out - thank you for all your advice today or if they ask for a letter from Home Office It will take them a long time to write a letter, I don’t know how to get one. Can you register me today anyway?
