# Supplementary material for: Evaluating adherence to patient registration paperwork guidelines: a mystery shopper study in English primary care
Source: BMJ Open. 2025 Nov 11;15(11):e100719. doi: 10.1136/bmjopen-2025-100719 (PMC13059835; doi:10.1136/bmjopen-2025-100719)
Supplement: Supplementary file 2 [file bmjopen-2025-100719.reviewer_comments.pdf]

PEER REVIEW HISTORY

BMJ Open publishes all reviews undertaken for accepted manuscripts. Reviewers are asked to complete a checklist review form and are provided with free text boxes to elaborate on their assessment. These free text comments are reproduced below.

ARTICLE DETAILS

Title (Provisional)

Evaluating Adherence to Patient Registration Paperwork Guidelines: A Mystery Shopper Study in English Primary Care

Authors

Hodson, Nathan; Onyeaso, Onyedikachi Oluferanmi; Mills, Stuart; Sunstein, Cass R.; Bruine de Bruin, Wändi

---

VERSION 1 - REVIEW

**Reviewer**	**1**
**Name**	**Sarkar, Amitabha**
**Affiliation**	**Graduate Institute of International and Development Studies, Global Health Centre**
**Date**	**05-May-2025**
**COI**	**None**

The paper makes an important contribution to the ongoing debate and discussion on NHS efficiency and effectiveness. It also adds value to the spirit of the right to health care in the contemporary world of disorder. The following are suggestions to further improve the paper.

While the background section begins with the NHS's founding principles and policy manuals, it lacks a short overview of the NHS's current state of affairs. This brief overview could set the context for why the study is needed, especially since the NHS as an institution is undergoing several crises.

The following line, "It is appropriate to characterise it [denial of care without Photo ID] as structurally racist allocation of care" in pg. no. 11, is not supported by the research results. Regarding the second research question on allowing registration without photo ID and proof of address over the phone, the paper already reported that the demographically homogeneous Herefordshire and Worcestershire region did not differ from the heterogeneous Birmingham and Solihull region in the advice given (χ^2 (1) = 0.70, p=0.15). In the absence of this difference, it is a far-fetched argument to claim that the denial of health care due to the unavailability of a photo ID is based on racial grounds.

The authors need to further review the NHS care modules. While the NHS guidelines state that a Photo ID is not mandatory for primary medical services, paragraph 4.9.4 of Part B in the Primary Medical Services Policy and Guidance Manual (PGM) also acknowledges that "when patients seek to register there is no regulatory requirement for patients to prove identity, address, immigration status or the provision of an NHS number in order to register. However, there are practical reasons why a practice might need to be assured that people are who they say they are, or to check where they live. Seeing some form of documentation regarding the patient's identity can help to ensure the correct matching of a patient to the NHS central patient registry, thereby ensuring any previous medical notes are passed onto a new practice. It is legitimate therefore for the practice to apply a consistent but non-discriminatory policy to ask for patient ID as part of their registration process" (https://www.england.nhs.uk/long-read/primary-medical-services-policy-and-guidance-manual-pgm/#part-b-general-contract-management). The authors need to understand that the NHS, as an institution, faces several bottlenecks, which knowingly or unknowingly create many trickle-down effects. For example, the authors may examine the NHS guidelines for the Identity Verification section, where identity verification by frontline health workers, including GPs, is part of the information governance and data protection protocol for medical records and patient registry (https://www.england.nhs.uk/long-read/identity-verification/). While the institution crumbles, its periphery often bears the impacts disproportionately.

The authors may review their analysis on pages 11–13, considering the above point. The authors' argument is to reorient the 'street-level bureaucrats' to remove the registration sludge. While providing care without photo ID proof upholds the right to health care principle, the authors also need to understand the bureaucratic hurdles frontline workers must circumvent in doing so.

The findings of the paper are highly important for advocating barrier-free access to health care and addressing health inequalities. The study findings also indicate the need to conduct a deeper analysis of frontline health workers in the context of NHS defunding. The paper may be accepted provided the authors give due consideration to the suggestions made in this review.

**Reviewer**	**2**
**Name**	**Lindenmeyer, Antje**
**Affiliation**	**University of Birmingham**
**Date**	**20-May-2025**
**COI**	**None**

This is an interesting study covering a very important topic. The abstract is clear and compares well with the main body text. The introduction cleary sets the scence and justifies

the research. It would be nice to also refer to the work of Doctors of the World UK in this area (e.g. the reports on Registration Refused (2015 and 2017).Methods are clear but there might be some discussion why these three were the only comparisons made (not a large enough sample to have smaller sub groups)? Also there could have been some discussion why this particular scenario was chosen (i.e. that person had a valid passport which was just temporarily unavailable) - would a scenario with an undocumented patient have lead to different response rates? Above you also mention a comparison between the two different 'mystery shoppers' - was this to explore potential discrimination?

Ethical approval is mentioned but using a 'mystery shopper' system like this, with some deception, should also be ethically justified.

The Findings session is a bit confusing. There is mention of website analysis looking for an explicit statement that ID was not needed to register, but numbers for this do not appear in Findings where practices are noted as 'following' or 'breaching' guidance on their websites. Would practices be categorised as 'following' guidance only if they have a clear statement in line with guidance or also if they do not explicitly exclude people without ID? Likewise, are they categorised as 'breaching' guidance when the first response was that ID was really needed or when the patient was unable to confirm they would be registered at the end of the discussion (perhaps with alternative ID)? And could the patient say as part of their script 'but on your website it says...' if there was divergence?

Also, if a lot more practices in H&W request ID on their website than in Birmingham, but there are no significant differences over the phone, does this mean that they were more likely in the category of practices where phone advice was more in line with guidance than their website (and any idea why)? It might be useful to have a version of Figure 3 separating out the two locations. It would also be nice to have a figure on the 'outcome' of the conversation if not identical with the 'breaching guidance in phone information' number (see above)

In the discussion, you say that the study shows that GPs and practice managers may be

unaware of the policy being implemented at their own practice (I assume by receptionists) - can this be included more explicitly in the findings? E.g. where did practice staff refer to 'practice policy' in the phone discussions? Similarly the statement "We identified GP

practices that stated on their websites that lacking proof of address would not exclude them

from registration. Yet at the same practices, callers were denied registration." could be fleshed out in Findings (see comments above).

Overall, the study is really valuable and I would like to see it published - however the Findings section could do with more detail and clarity.

NB I couldn't find a statement on funding - the STROBE statement said it was on p11!

VERSION 1 - AUTHOR RESPONSE

Reviewer: 1<br>Dr. Amitabha Sarkar, Graduate Institute of International and Development Studies<br>Comments to the Author:	
The paper makes an important contribution to the ongoing debate and discussion on NHS efficiency and effectiveness. It also adds value to the spirit of the right to health care in the contemporary world of disorder. The following are suggestions to further improve the paper.	Thank you Dr Sarkar for taking the time to review this manuscript. We are grateful for your comments.
While the background section begins with the NHS's founding principles and policy manuals, it lacks a short overview of the NHS's current state of affairs. This brief overview could set the context for why the study is needed, especially since the NHS as an institution is undergoing several crises.	Thank you we agree with the reviewer about the connection between this context and the paperwork.<br><br>We have addressed the point raised in the discussion section in the paragraph "To be sure, the NHS, especially primary care, is under extraordinary pressure…".<br><br>On balance, we believe that the discussion is the appropriate place to address this topic.

The following line, "It is appropriate to characterise it [denial of care without Photo ID] as structurally racist allocation of care" in pg. no. 11, is not supported by the research results. Regarding the second research question on allowing registration without photo ID and proof of address over the phone, the paper already reported that the demographically homogeneous Herefordshire and Worcestershire region did not differ from the heterogeneous Birmingham and Solihull region in the advice given (χ^2 (1) = 0.70, p=0.15). In the absence of this difference, it is a far-fetched argument to claim that the denial of health care due to the unavailability of a photo ID is based on racial grounds.	We are grateful for the reviewer's comments on this.<br><br>Our point was not that there was a difference between regions, but that any policy which excludes people based on photo ID is disproportionately impacting Black British people.<br><br>We are grateful for the opportunity to make this points more clearly and have added the following explanation:<br><br>"While our findings do not reveal any intent on the part of GP practice staff to single people out by race, the widespread requirement for ID and

	proof of address may exclude many more Black British people than White British people.[9]”
The authors need to further review the NHS care modules. While the NHS guidelines state that a Photo ID is not mandatory for primary medical services, paragraph 4.9.4 of Part B in the Primary Medical Services Policy and Guidance Manual (PGM) also acknowledges that “when patients seek to register there is no regulatory requirement for patients to prove identity, address, immigration status or the provision of an NHS number in order to register. However, there are practical reasons why a practice might need to be assured that people are who they say they are, or to check where they live. Seeing some form of documentation regarding the patient’s identity can help to ensure the correct matching of a patient to the NHS central patient registry, thereby ensuring any previous medical notes are passed onto a new practice. It is legitimate therefore for the practice to apply a consistent but non-discriminatory policy to ask for patient ID as part of their registration process” (https://www.england.nhs.uk/long-read/primary-medical-services-policy-and-guidance-manual-pgm/#part-b-general-contract-management). The authors need to understand that the NHS, as an institution, faces several bottlenecks, which knowingly or unknowingly create many trickle-down effects. For example, the authors may examine the NHS guidelines for the Identity Verification section, where identity verification by frontline health workers, including GPs, is part of the information governance and data protection protocol for medical records and patient registry (https://www.england.nhs.uk/long-read/identity-verification/). While the institution crumbles, its periphery often bears the impacts disproportionately. The authors may review their analysis on pages 11–13, considering the above point. The authors’ argument is to reorient the ‘street-level bureaucrats’ to remove the registration sludge. While providing care without photo ID proof upholds the right to health care principle, the authors also need to understand the bureaucratic hurdles frontline workers must circumvent in doing so.	Thanks for this food for thought.<br><br>We understand that the NHS is facing pressures and two of the authors are practicing doctors in the NHS. We will not add further discussion of the NHS ‘crumbling’ or ‘in crisis’.<br><br>We have addressed that in this paragraph:<br><br>“To be sure, the NHS, especially primary care, is under extraordinary pressure. But in this context, registration sludge is not an acceptable way to ration care, as NHS England itself has said.[26] Not only is the relevant sludge unfair and opaque but the additional administrative work involved means that even among people who have all required paperwork, there is a risk that healthcare will be delayed.[27] Alternatively, the patient may take the advice given at several practices and use 111 or urgent care instead, meaning their care costs the NHS more and lacks the continuity primary care provides. Interventions to tackle this sludge could realise efficiencies in primary care provision while expanding primary care coverage.”<br><br>We wish to address the point about a purported inconsistency in the NHS PGM. In fact, the guidance does allow practices to ask for patient ID, but it does not allow practices to refuse registration based on the lack of ID.

	The mystery shoppers were refused registration. They explained that they lacked ID and they asked for help. They were told that they could not be registered.<br><br>We have therefore added the following paragraph to address the reviewer's important point:<br><br>"We note that there is a subsequent line in the NHS PGM stating that practices may "apply a consistent but non-discriminatory policy to ask for patient ID" during the registration process (Part B 4.9.4).[2] Some might contend that this contradicts the clear instruction in the NHS PGM that "If a patient cannot produce any supportive documentation but states that they reside within the practice boundary then practices should accept the registration" because many people are "legitimately unable to produce any of the listed documentation."[2] These lines are not contradictory: practices may 'ask for patient ID' but if the patient cannot provide it then practices should register the patient anyway. What this study measured was whether registration was possible without ID, not whether ID was requested. Indeed, in cases where ID was requested but not required and registration was possible without it, the practice was recorded as in keeping with guidance."<br><br>Again, thank you for the opportunity to address this interesting point.
The findings of the paper are highly important for advocating barrier-free access to health care and addressing health inequalities. The study findings also indicate the need to conduct a deeper analysis of	Thank you again for these comments.

frontline health workers in the context of NHS defunding. The paper may be accepted provided the authors give due consideration to the suggestions made in this review.	
Reviewer: 2<br>Dr. Antje Lindenmeyer, University of Birmingham<br>Comments to the Author:	
This is an interesting study covering a very important topic. The abstract is clear and compares well with the main body text. The introduction cleary sets the scence and justifies the research.	We are very grateful to Dr Lindenmeyer for these thoughtful comments.
It would be nice to also refer to the work of Doctors of the World UK in this area (e.g. the reports on Registration Refused (2015 and 2017).	Thank you for these helpful references which have been added, along with this text:<br><br>"Indeed, in the 2010s several studies identified that proof of address was leading to many people being wrongly denied registration at a GP practice. Doctors of the World UK reported that among eligible people they supported to register with a GP practice, the vast majority were asked for photo ID, but with support and advocacy from Doctors of the World UK, these requirements were overcome in 48% of cases.[15] While it is known that ID requests are widespread, it is unknown how many patients would be able to register without the active intervention of a charity like Doctors of the World UK, which is not available in most cases.[5, 16] "
Methods are clear but there might be some discussion why these three were the only comparisons made (not a large enough sample to have smaller sub groups)?	Yes, we have added this to the methods (analyses) section:<br><br>"Subgroup analyses were conducted but interpreted with caution due to low numbers."
Also there could have been some discussion why this particular scenario was chosen (i.e. that person had a valid passport which was just temporarily unavailable) -	Thank you we have added an explanation of why:

would a scenario with an undocumented patient have lead to different response rates?	"This script was chosen because it included a common reason why people do not have a GP or photo ID, and it also explained why the mystery shopper did not have any bills in his own name."
Above you also mention a comparison between the two different 'mystery shoppers' - was this to explore potential discrimination?	We realised it was necessary to check whether there was any difference in the treatment of the two mystery shoppers who could appear quite different over the phone so we checked whether there was any difference and reported that none was found. ($\chi^2(1)=0.01$, $p=0.91$).
Ethical approval is mentioned but using a 'mystery shopper' system like this, with some deception, should also be ethically justified.	We have added the following explanation,<br><br>"Ethical approval was provided by the Institutional Review Board of the University of Southern California. The deception inherent to this mystery shopper study was ethically defensible because (1) non-deceptive research methods such as interviews or surveys with practice staff may have underestimated violation of NHS guidance due to social desirability bias, (2) the importance of access to healthcare, especially among vulnerable populations without photo ID or proof of address, (3) the confidentiality of GP staff, and (4) the limited time burden on GP staff. [24]."
The Findings session is a bit confusing. There is mention of website analysis looking for an explicit statement that ID was not needed to register, but numbers for this do not appear in Findings where practices are noted as 'following' or 'breaching' guidance on their websites. Would practices be categorised as 'following' guidance only if they have a clear statement in line with guidance or also if they do not explicitly exclude people without ID? Likewise, are they categorised as 'breaching' guidance when the first response was that ID was really	Yes, we have now clarified in the text. For the websites:<br><br>"Practices were coded as breaching the guidance if they stated that ID was required. They were coded as following the guidance if they did not state ID was required or if they stated that ID was not required."

needed or when the patient was unable to confirm they would be registered at the end of the discussion (perhaps with alternative ID)?	And over the phone:<br><br>"Practices were coded as breaching the guidance if they insisted that ID was required despite finding out that the patient could not provide ID. They were coded as following the guidance if at any point they stated that ID was not required."
And could the patient say as part of their script 'but on your website it says...' if there was divergence?	No the patient's scripts are given in the appendix and they could not state this, nor the NHS guidance.<br><br>We have now added a sentence about this:<br><br>"Mystery shoppers did not refer back to the website or the NHS guidance during the conversation."
Also, if a lot more practices in H&W request ID on their website than in Birmingham, but there are no significant differences over the phone, does this mean that they were more likely in the category of practices where phone advice was more in line with guidance than their website (and any idea why)? It might be useful to have a version of Figure 3 separating out the two locations. It would also be nice to have a figure on the 'outcome' of the conversation if not identical with the 'breaching guidance in phone information' number (see above)	Thanks for raising this interesting point. We have added this comment: "Rates of consistency between website and telephone advice did not differ between the Birmingham and Solihull region and the Herefordshire and Worcestershire region ($\chi^2(1) = 1.57, p=0.21$)." We felt that a figure reporting this non-significant finding could be misleading so we have not added a figure, but agree that adding this comment is worthwhile.
In the discussion, you say that the study shows that GPs and practice managers may be<br>unaware of the policy being implemented at their own practice (I assume by receptionists) - can this be included more explicitly in the findings? E.g. where did practice staff refer to 'practice policy' in the phone discussions?	Thanks we have included this point more explicitly in the manuscript:<br><br>"GP partners and practice managers should keep in mind that, whatever information is written on the website, an exclusionary policy may be implemented by receptionists when prospective patients contact the practice"

Similarly the statement "We identified GP practices that stated on their websites that lacking proof of address would not exclude them<br>from registration. Yet at the same practices, callers were denied registration." could be fleshed out in Findings (see comments above).	Thank you we have fleshed this out with the following statement: “Thus, practices are publicly stating a sludge-free policy online whilst implementing an exclusionary policy over the phone, perhaps unbeknownst to GPs and practice managers. This sludge is opaque and difficult for activists and scholars to challenge given the most common way of evaluating policies has been through website reviews.”
Overall, the study is really valuable and I would like to see it published - however the Findings section could do with more detail and clarity.	Thanks for taking the time to consider this manuscript and for sharing these valuable comments which have strengthened the manuscript.
NB I couldn't find a statement on funding - the STROBE statement said it was on p11!	Thanks! Added to page 8 now.

VERSION 2 - REVIEW

**Reviewer**	**2**
**Name**	**Lindenmeyer, Antje**
**Affiliation**	**University of Birmingham**
**Date**	**16-Jul-2025**
**COI**	

All my concerns have been addressed and I would be happy to see this paper published.
